# Novel macrolide-lincosamide-streptogramin B resistance gene *erm*(56) in *Trueperella pyogenes*

**DOI:** 10.1128/msphere.00239-23

**Published:** 2023-07-07

**Authors:** Emma Marchionatti, Vincent Perreten

**Affiliations:** 1 Division of Molecular Bacterial Epidemiology and Infectious Diseases, Institute of Veterinary Bacteriology, Vetsuisse Faculty, University of Bern, Bern, Switzerland; 2 Clinic for Ruminants, Department of Clinical Veterinary Science, Vetsuisse Faculty, University of Bern, Bern, Switzerland; University of Nebraska Medical Center, Omaha, Nebraska, USA

**Keywords:** macrolides-lincosamides-streptogramin B, mechanisms of resistance, DNA sequencing, gene expression

## Abstract

Whole-genome sequence analysis of a macrolide, lincosamide, streptogramin B (MLS_B_)-resistant *Trueperella pyogenes* from a dog revealed a new 23S ribosomal RNA methylase gene *erm*(56). Expression of the cloned *erm*(56) confers resistance to MLS_B_ in *T. pyogenes* and *Escherichia coli*. The *erm*(56) gene was flanked by two IS*6100* integrated on the chromosome next to a *sul1*-containing class 1 integron. GenBank query revealed additional *erm*(56)-containing elements in another *T. pyogenes* and in *Rothia nasimurium* from livestock.

**IMPORTANCE:**

A novel 23S ribosomal RNA methylase gene *erm*(56) flanked by insertion sequence IS*6100* was identified in a *Trueperella pyogenes* isolated from the abscess of a dog and was also present in another *T. pyogenes* and in *Rothia nasimurium* from livestock. It was shown to confer resistance to macrolide, lincosamide, streptogramin B antibiotics in *T. pyogenes* and *E. coli,* indicating functionality in both Gram-positive and Gram-negative bacteria. The detection of *erm*(56) on different elements in unrelated bacteria from different animal sources and geographical origins suggests that it has been independently acquired and likely selected by the use of antibiotics in animals.

## OBSERVATION

*Trueperella pyogenes*, a commensal Gram-positive bacterium of the skin and mucous membranes of animals, can cause suppurative infections in multiple animal species and rarely humans (1). Despite macrolides and lincosamides being antibiotics used as second-line treatment for these infections, their usage may contribute to the selection of antimicrobial resistances. So far, acquired resistances to macrolide, lincosamide, and streptogramin B (MLS_B_) antibiotics in *T. pyogenes* have been associated with the presence of erythromycin ribosome methylase (*erm*) genes, specifically *erm*(B) and *erm*(X), that prevent the binding of the MLS_B_ antibiotics to the 23S rRNA (2 - 4). In *T. pyogenes*, these genes have been reported within mobile genetic elements as either interposed between insertion sequence (IS) elements (5) or integrated in a class 1 integron together with other antimicrobial resistance genes (6).

### Detection and characterization of *erm*(56)

**Fig 1:**
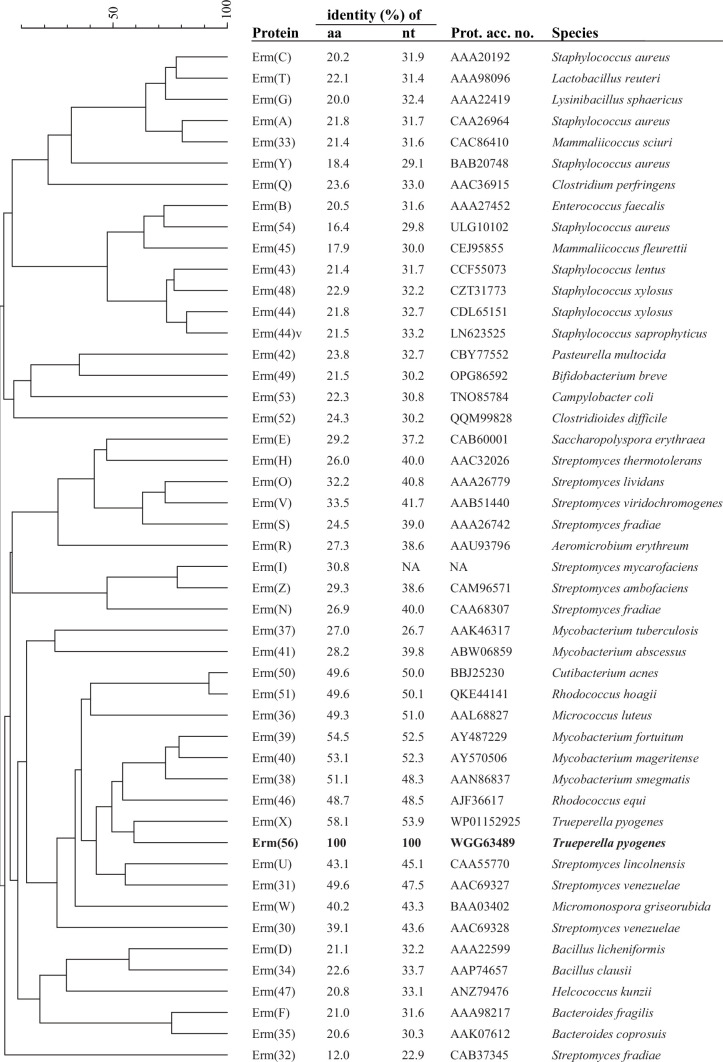
Relationship tree of all known Erm methylases, including the novel Erm(56) detected in *T. pyogenes* 09KM1269. Amino acid (aa) and nucleotide (nt) identity was obtained by sequence alignment using Clustal Omega 1.2.2 (Geneious Prime 2022.2.2, Biomatters Ltd., Auckland, New Zealand). The tree was constructed using BioNumerics 8.1.1 (BioMérieux, Marcy-l'Étoile, France) and the following settings: standard algorithm for pairwise alignment; open gap penalty, 100%; unit gap penalty, 0%; and unweighted pair group method using average linkages. The protein sequences are indicated by their GenBank accession numbers. The Erm(I) amino acid (aa) sequence is not available (NA) in the GenBank and was obtained from its original publication (13).

*T. pyogenes* strain 09KM1269, isolated from an abscess of a dog in 2009 in Switzerland, exhibited constitutive resistance to erythromycin and clindamycin as determined by Clinical and Laboratory Standards Institute (CLSI) criteria (7), suggesting the presence of an MLS_B_ methylase (Erm) (Table 1). The absence of *erm*(B) and *erm*(X) as determined using previously described PCR assays (8) prompted us to search for the underlying resistance mechanism by whole-genome sequence analysis. Genomic DNA was extracted using MasterPure Complete DNA and RNA Purification Kit (Lucigen, Middleton, WI), sequenced on a PacBio Sequel IIe system (Next-Generation Sequencing Platform, University of Bern), and the resulting reads were *de novo* assembled using Flye 2.9.1 (9). Analysis of the complete genome using ResFinder 4.1 (Center for Genomic Epidemiology, Denmark) and blast search showed the absence of any so-far known *erm* gene listed in the Nomenclature Center for MLS_B_ Genes (https://faculty.washington.edu/marilynr/) (10). GenBank query using tblastx (https://blast.ncbi.nlm.nih.gov) and *erm*(X) as subject sequence (GenBank accession number NC_005206) identified a novel putative *erm* gene. This gene encoded a 267-aa 23S rRNA methylase and showed the closest relatedness to the Erm(X) determinant of plasmid pAP2 from *T. pyogenes* with 58% amino acid (aa) and 54% nucleotide (nt) identity (Fig. 1) and was designated *erm*(56) (http://faculty.washington.edu/marilynr/) (10). Putative -35 (TTGACC) and -10 (TGCTAATGT) promoter sequences were identified using BPROM (11) 31 and 11 bp upstream of the putative guanine transcription start located 153 bp upstream of *erm*(56). This 153 bp upstream region contained the ribosomal binding sites and imperfect inverted repeats capable of folding into stem-loops which may play a role in the translational attenuation of the Erm(56) methylase (12) (Fig. S1).

**TABLE 1:** MIC of erythromycin, clindamycin, pristinamycin IA, and pristinamycin IIA for different *T. pyogenes* and *E. coli* strains, as determined by broth microdilution

Strain	Characteristic(s) or origin	Reference or source	Antibiotic resistance gene(s)^*a*^	MIC (μg/mL)^*b*^			
				ERY	CLI	PIA	PIIA
*T. pyogenes* 09KM1269	Dog abscess sample	This study	*erm*(56), *sul1*	>256	128	32	≤0.25
*E. coli* AG100A	Recipient strain, plasmid free, Δ*acrAB*::KAN^R^	Reference 14	*aph(3')-II*	2	2	128	2
*T. pyogenes* 13OD0707	Recipient strain, plasmid free	This study		≤0.25	≤0.25	2	≤0.25
13OD0707/pJRD215	13OD0707 with cloning vector pJRD215 (KAN^R^)	This study	*aph(3')-II*	≤0.25	≤0.25	2	≤0.25
13OD0707/pJEM1269	13OD0707 with *erm*(56) and promoter region from *T. pyogenes* 09KM1269 cloned into pJRD215	This study	*erm*(56)*, aph(3')-II*	>256	128	32	≤0.25
AG100A/pJEM1269	AG100A with *erm*(56) and promoter region from *T. pyogenes* 09KM1269 cloned into pJRD215	This study	*erm*(56)*, aph(3')-II*	128	16	128	128

^*a*^ Antibiotic resistance genes and functions: *erm*(56), 23S rRNA methylase; *sul1*, sulfonamide-resistant dihydropteroate synthase; *aph(3')-II*, aminoglycosides phosphotransferase.

^*b*^ ERY, erythromycin; CLI, clindamycin; KAN, kanamycin; PIA, pristinamycin IA; PIIA, pristinamycin IIA.

### Cloning and expression of *erm*(56)

To test the functionality of *erm*(56), a 1,078 bp region of strain 09KM1269 including *erm*(56) and promoter sequences was amplified by PCR using Q5 High-Fidelity DNA Polymerase (New England Biolabs, MA, USA) and primers erm56-SalI-F3 (5′-cacatgtcgacGCCATCACACTGCTTGTTTCAACGA) and erm56-SpeI-R (5′-cacagactagtATTTTTCTTGGCCCGCCTCCCCAGA) (annealing temperature, 58°C; extension time, 1 min). The primers contained overhangs (lowercase) with restriction site sequences (underlined) to facilitate cloning into the SalI and SpeI restriction sites of pJRD215 (15). The resulting *erm*(56)-containing plasmid pJEM1269 was obtained in *Escherichia coli* DH5α after ligation and heat shock transformation and selection on LB agar plates containing 30 µg/mL kanamycin. Plasmid pJEM1269 was subsequently transformed by electroporation into susceptible strains of *E. coli* AG100A (Δ*acrAB*::KAN^R^) (14) using electroporation cuvettes of 0.1 cm and settings of 2.3 kV/cm, 25 µF, 200 Ω and of *T. pyogenes* 13OD0707 (GenBank accession number CP123403) using settings of 18 kV/cm, 50 µF, 246 Ω and a time constant of 10 ms (16). *E. coli* AG100A and *T. pyogenes* 13OD0707 electrotransformants were selected on LB containing 10 µg/mL erythromycin and BHI agar containing 5% sheep blood and 30 µg/mL kanamycin, respectively.

MIC values of erythromycin (macrolide), clindamycin (lincosamide) (Sigma-Aldrich, St-Louis, MO, USA), pristinamycin IA (streptogramin B), and pristinamycin IIA (streptogramin A) (Molcan Corporation, Richmond Hill, ON, Canada) of *E. coli* and *T. pyogenes* strains were determined by broth microdilution using Mueller-Hinton broth supplemented with 5% lysed horse blood and 48 h incubation for *Trueperella* following CLSI recommendations (7) (Table 1). When *erm*(56) was expressed from plasmid pJEM1269 in *T. pyogenes* 13OD0707, the MIC of MLS_B_ antibiotics increased by more than 256-fold for erythromycin and clindamycin and by 16-fold for pristinamycin IA, while no difference was seen for the streptogramin A pristinamycin IIA as assumed for an Erm methylase. Increased MICs of erythromycin (64-fold) and clindamycin (8-fold) were also measured for AG100A containing pJEM1269, but no conclusion could be drawn for pristinamycin IA, for which the MIC was already high for the recipient strain and remained unchanged in the presence of *erm*(56) (Table 1).

### Genomic location of *erm*(56) and detection in additional bacteria

**Fig 2:**
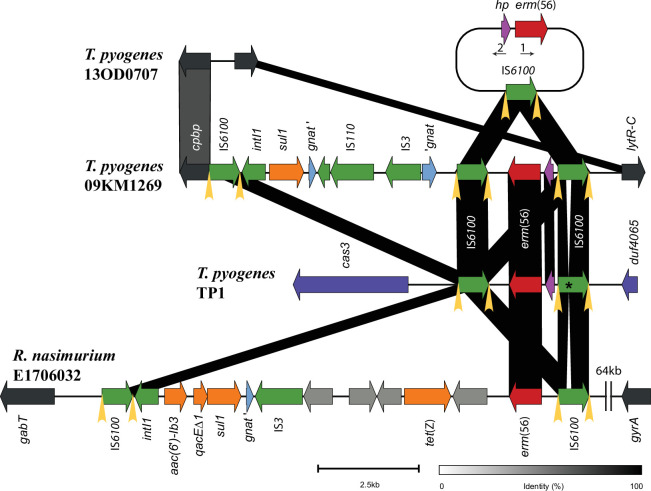
Schematic gene map showing the *erm*(56)-containing elements and flanking region of *T. pyogenes* 09KM1269 (GenBank accession number CP123393) compared to the chromosomal region of *erm*(56)-negative strain 13OD0707 (GenBank accession number CP123403), as well as the integration of *erm*(56) in *T. pyogenes* TP1 (GenBank accession number CP033902) and *R. nasimurium* E1706032 (GenBank accession number CP056080). Black and dark-gray areas represent regions showing 100% and 85% similarity at nucleotide level, respectively. Arrows represent open reading frames (ORFs). The ORF of *erm*(56) is indicated by a red arrow, and the *erm*(56)-preceding ORF (hypothetical protein gene, *hp*) is in purple. ORFs of other antibiotic resistance genes are shown in orange: *sul1*, dihydropteroate synthase gene for sulfonamide resistance; *aac(6')-Ib3*, aminoglycoside *N*-acetyltransferase gene; *qacEΔ1*, quaternary ammonium compound efflux transporter gene; *tet*(Z), tetracycline efflux transporter gene. Other ORFs of hypothetical proteins are represented by gray arrows. ORFs of transposase genes associated with IS and integrase (*intI1*) are indicated with green arrows. Asterisk (*) within IS*6100* of *T. pyogenes* TP1 indicates a cytosine deletion at position 164 leading to a frameshift of the transposase of IS*6100*. ORFs of phage protein genes are indicated in dark blue. The 3′-end truncated part (*gnat*′) and the 5′-end truncated part (′*gnat*) of the GNAT *N*-acetyltransferase gene are indicated in light blue. ORFs of core genome genes are indicated as dark gray arrows. Inverted repeats of the IS*6100* elements are indicated by yellow arrow heads (IR-L, GGCTCTGTTGCAAA; IR-R, TTTGCAACAGAGCC). Circular conformation of the IS*6100-erm*(56) element obtained by PCR using primers erm56(159)-F and hp-erm56(45)-R (indicated by small arrows 1 and 2) is represented by a circle containing the respective ORFs. The figure was generated using Clinker (18) and Adobe Illustrator.

The *erm*(56) gene of *T. pyogenes* 09KM1269 was preceded by a hypothetical protein (hp) and integrated into the chromosome between two identical IS*6100* elements located in the same orientation, each flanked by 14 bp inverted repeats (IR-L, GGCTCTGTTGCAAA; IR-R, TTTGCAACAGAGCC). The IS*6100*-hp-*erm*(56)-IS*6100* element was situated next to a class 1 integron containing the sulfonamide resistance gene *sul1*, which was also delimited by a copy of IS*6100* (Fig. 2). GenBank searches also identified *erm*(56) associated with IS*6100* in *T. pyogenes* strain TP1 isolated from bovine lung in China (GenBank accession number CP033902) and in *Rothia nasimurium* strain E1706032 isolated from duck brain in China (GenBank accession number CP056080) (Fig. 2). In *T. pyogenes* TP1, the IS*6100*-hp-*erm*(56)-IS*6100* element was found on a large 27.4 kb fragment which was related to the *Lactobacillus* phage PLE2 (GenBank accession number NC031036) using PHASTER (17). The sequence of the IS*6100* situated upstream of *erm*(56) in *T. pyogenes* TP1 had a cytosine deletion at position 164 leading to a frameshift and an early stop codon in the IS*6100* transposase. In *R. nasimurium* E1706032, *erm*(56) was only preceded by one IS*6100* and integrated next to *tet*(Z) and a class 1 integron containing *aac(6')-Ib3*, *qacEΔ1,* and *sul1*; this resistance element was delimited by two copies of IS*6100* (Fig. 2).

Although a circular conformation containing one copy of IS*6100* was detected by PCR using primers pointing outward from *erm*(56) [erm56(159)-F, 5′-GGGACGATCTCTCACAGCTG and hp-erm56(45)-R, 5′-GAGAACTCGACCCAAACGAGGATCA; annealing temperature, 60°C; extension time, 2 min] and subsequent Sanger sequencing (Fig. 2), the *erm*(56) gene could not be transferred by either filter mating (19) into MLS_B_ susceptible and plasmid-free *T. pyogenes* strain 13KM1326 (STR^R^; RpsL mutation K43R) (GenBank accession number CP123400) or electroporation of genomic DNA (16) into *T. pyogenes* 13OD0707 (GenBank accession number CP123403) using BHI agar containing 5% sheep blood (BHI-S) added of 50 µg/mL of streptomycin and 10 µg/mL of erythromycin for the selection of transconjugants and 10 µg/mL of erythromycin for the selection of electrotransformants.

Although *erm*(56) could not be transferred *in vitro*, its detection on different elements in unrelated bacteria suggests that it has a potential for broader dissemination. Given its detection in bacteria from different animal sources and geographical origins, it is likely that it has been independently selected by the use of antibiotics in animals.

## Data Availability

The nucleotide sequence of *erm*(56) of *T. pyogenes* 09KM1269 was deposited into the GenBank/ENA/DDBJ databases under accession number OQ326498. The complete genome sequences of *T. pyogenes* 09KM1269, 13OD0707, and 13KM1326 were deposited into the GenBank under accession numbers CP123393, CP123403, and CP123400.

## SUPPLEMENTAL MATERIAL

Figure S1 msphere.00239-23-s0001.pdf Schematic minimum free energy (MFE) structure encoding base pair probabilities and predicting secondary structure of the 153-bp upstream DNA sequence upstream of the start codon of *erm*(56).

ASM does not own the copyrights to Supplemental Material that may be linked to, or accessed through, an article. The authors have granted ASM a non-exclusive, world-wide license to publish the Supplemental Material files. Please contact the corresponding author directly for reuse.
