# Supplementary material for: Novel macrolide-lincosamide-streptogramin B resistance gene erm(56) in Trueperella pyogenes
Source: mSphere. 2023 Jul 7;8(4):e00239-23. doi: 10.1128/msphere.00239-23 (PMC10470526; doi:10.1128/msphere.00239-23)
Supplement: Figure S1 — Schematic minimum free energy (MFE) structure encoding base pair probabilities and predicting secondary structure of the 153-bp upstream DNA sequence upstream of the start codon of erm(56). [file msphere.00239-23-s0001.pdf]

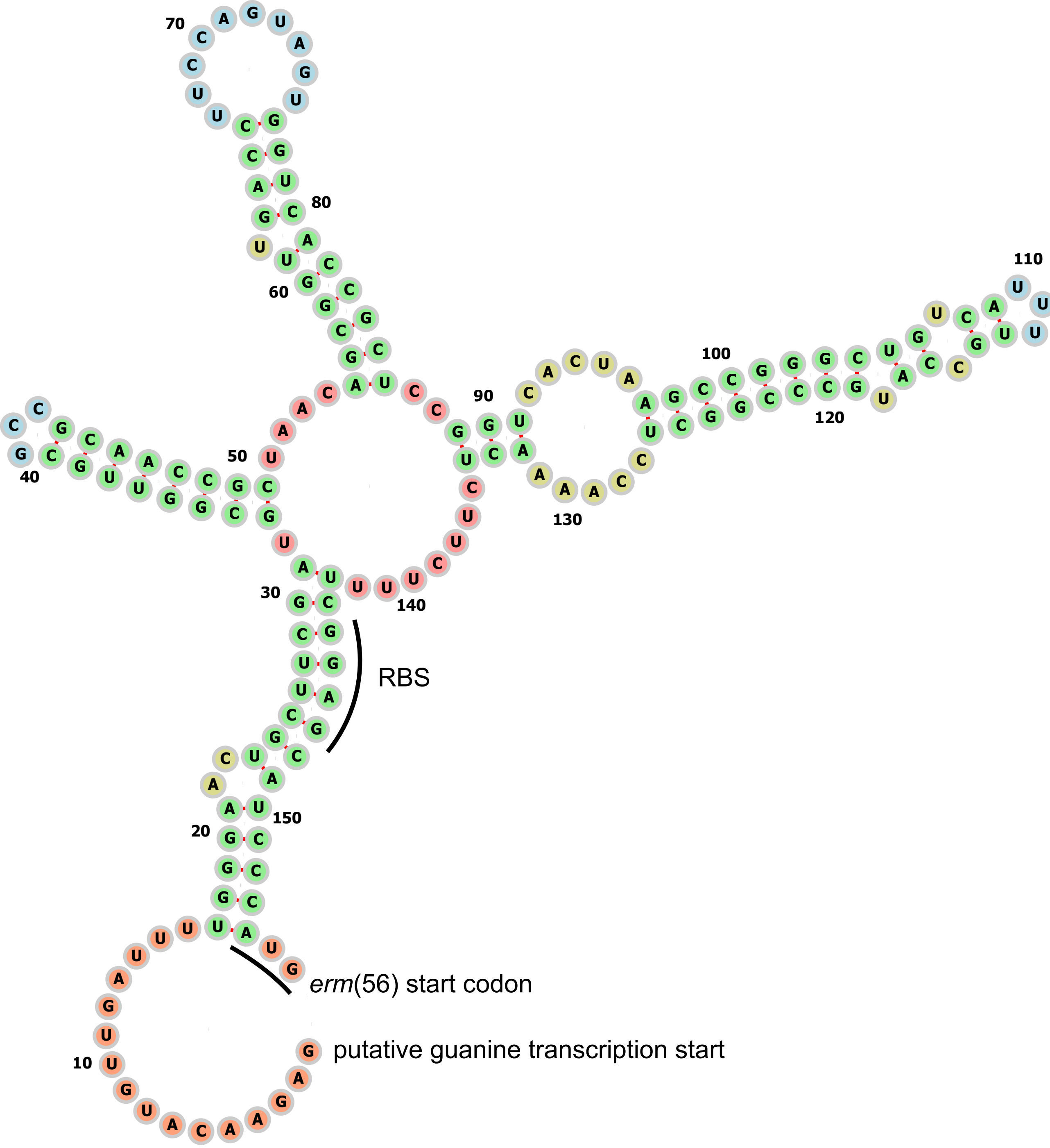

Supplementary Figure S1. Schematic minimum free energy (MFE) structure encoding base-pair probabilities and predicting secondary structure of the 153-bp upstream DNA sequence upstream of the start codon of *erm*(56). RBS, ribosomal binding site of *erm*(56). The type of structure formed by the nucleotides are indicated with different colors: Green, stems (canonical helices); Red; multi-loops (junctions); Yellow, interior loops; Blue: hairpin loops; Orange: 5' and 3' unpaired region. The figure was generated using the RNAfold web server (Gruber AR et al. The Vienna RNA websuite. Nucleic Acids Res. 2008 Jul 1;36(Web Server issue):W70-4).
